# Aroclor 1254 induced inhibitory effects on osteoblast differentiation in murine MC3T3-E1 cells through oxidative stress

**DOI:** 10.3389/fendo.2022.940624

**Published:** 2022-10-24

**Authors:** Yu Chen, Yuwei Cai, Chunxiang Chen, Mengting Li, Lingdan Lu, Zhongxiang Yu, Shuqiang Wang, Lei Fang, Shengming Xu

**Affiliations:** ^1^ Department of Orthopaedic Surgery (I), Shuguang Hospital, Shanghai University of Chinese Traditional Medicine, Shanghai, China; ^2^ Department of Neurology, Yueyang Hospital of Integrated Chinese and Western Medicine Affiliated to Shanghai University of Traditional Chinese Medicine, Shanghai, China; ^3^ Department of Orthopaedic Surgery, Yueyang Hospital of Integrated Chinese and Western Medicine Affiliated to Shanghai University of Traditional Chinese Medicine, Shanghai, China

**Keywords:** MC3T3-E1 cell, Aroclor 1254, oxidative stress, calcium homeostasis, osteoblast differentiation

## Abstract

This study aimed to evaluate the osteotoxicity of polychlorinated biphenyls in murine osteoblastic MC3T3-E1 cells, and to explore the underlying mechanism focused on oxidative stress. The cells were exposed to Aroclor 1254 at concentrations of 2.5-20 µmol/L, and then cell viability, oxidative stress, intracellular calcium concentration, osteocalcin content, and calcium nodules formation were measured. Aroclor 1254 reduced cell viability and induced overproduction of intracellular reactive oxygen species in a dose-dependent manner. Activity of superoxide dismutase was decreased, and malondialdehyde content was promoted after exposure. Moreover, inhibitory effects of Aroclor 1254 on calcium metabolism and mineralization of osteoblasts were observed, as indicated by reduction of the intracellular calcium concentration, osteocalcin content, and modules formation rate. The decreased expression of osteocalcin, alkaline phosphatase, bone sialoprotein, and transient receptor potential vanilloid 6 further confirmed the impairment of Aroclor 1254 on calcium homeostasis and osteoblast differentiation. Addition of the antioxidant N-acetyl-L-cysteine partially restored the inhibitory effects on calcium metabolism and mineralization. In general, Aroclor 1254 exposure reduces calcium homeostasis, osteoblast differentiation and bone formation, and oxidative stress plays a vital role in the underlying molecular mechanism of osteotoxicity.

## Graphical Abstract

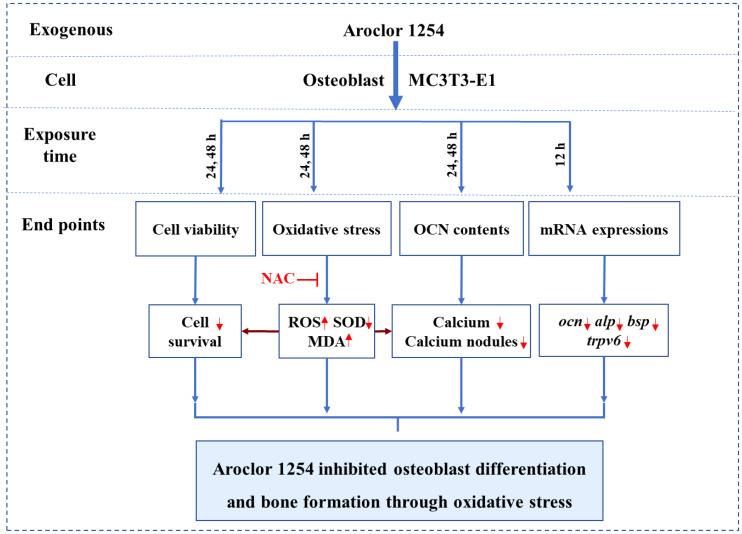

## Introduction

Due to the excellent electrical insulation and heat resistance, polychlorinated biphenyls (PCBs) had been extensively applied as insulating agents, lubricants, special industrial additives for approximately 50 years until 1980 (1–3). It is estimated that more than 1 million tons of PCBs have been produced and used, of which 1/4 to 1/3 has entered the environment. Although many developed countries have restricted the use of PCBs or substituted them by materials with lower toxicity, large amounts of discarded PCBs are still widely present in the environment (4, 5). PCBs can be accumulated in environmental media and be biomagnified through the food chain because of their bioaccumulation potential (3). Furthermore, PCBs are stable and resistant to environmental degradation, so that they can persist in both the environment and living organisms (3, 4). Despite the implement of relevant control measures on PCBs since decades ago, the total PCBs concentrations in air and dust reach microgram levels per cubic meter in severe-pollution area (6, 7). It is reported that the internal exposure concentrations of PCBs range from 99 to 2152 ng/g lipid (Median: 454 ng/g lipid) in the whole blood samples from German adults, and 52 to 933 ng/g lipid (Median: 226 ng/g lipid) in plasma samples (8).

As persistent organic pollutants identified by the Stockholm Convention, the toxicity of PCBs has received worldwide attention. It has been proven that PCB exposure is associated with the developmental neurotoxicity, endocrine dysfunction, and liver dysfunction (9–11). In addition, PCBs competitively bind to estrogen or androgen receptors, interfering with the estrogen/androgen balance (12, 13). In 2016, PCBs were classified as human carcinogens by the International Agency for Research on Cancer (14).

Hodgson etal. (15) performed an epidemiological study in local residents living near the Baltic coast in Sweden, who might experience PCB exposure through PCB-contaminated fish or water due to an upstream paper mill. Their results revealed that PCB exposure was associated with lowered bone mineral density in a sex-specific manner. PCB105/PCB118 exposure reduced the stiffness index of bone quality/strength in Cree women of the Eastern James Bay (Canada) (16). Aroclor 1254 (A1254) is a commercial PCB mixture with biphenyl and 54% chlorine, which is commonly used in the toxicology research. A1254 contains more than 60 PCB congeners, representing the dominant congeners present in environmental samples such as PCB47, PCB153 and PCB77 (17, 18). Ramajayam etal. (19) found that A1254 exposure disrupted the femoral bone metabolism of adult male Wistar rats through stimulating oxidative stress. Johnson etal. (20) also reported that PCB exposure impaired the bone mineral density in deer mice (*Peromyscus maniculatus*). Perinatal exposure tests in Sprague-Dawley rat offspring showed that A1254 could disrupt bone geometry and reduce mineral density, resulting in shorter, thinner, and weaker bones (21). However, the molecular mechanisms responsible for the osteotoxicity induced by PCB exposure have not yet been clarified.

Calcium is an essential structural component of the skeleton, and disruption of the calcium balance is recognized as one of the major causes for bone metabolism deterioration (22). Oxidative stress plays a crucial role in the occurrence of age-related osteoporosis through disrupting the balance between bone resorption and formation (23). Once excessive generation of free radicals overwhelms the scavenging ability of the natural antioxidants defense system, hyperoxidant stress leads to calcium imbalance (24, 25). It was reported that oxidative stress might promote osteoporosis in rats through the receptor activator of the nuclear factor kappa-B ligand/osteoprotegerin pathway (26). Furthermore, oxidative stress was confirmed to be one of the potential underline mechanisms for PCB-induced hepatotoxicity and neurotoxicity (27, 28). However, the role of oxidative stress on bone differentiation and formation after PCB exposure is not fully understood.

In the present work, the oxidation induction of A1254 in MC3T3-E1 cells was evaluated through detecting the reactive oxygen species (ROS) production, superoxide dismutase (SOD) activity, and malondialdehyde (MDA) level. Calcium metabolism and mineralization of osteoblasts were evaluated by intracellular calcium ion concentration, osteocalcin (OCN) content, and calcium nodule formation. At the same time, the related molecular markers of bone differentiation such as OCN, alkaline phosphatase (ALP), bone sialoprotein (BSP), and transient receptor potential vanilloid 6 (TRPV6) were detected by quantitative reverse transcription polymerase chain reaction (RT-qPCR). To clarify the role of oxidative stress in PCB-induced osteotoxicity, the antioxidant N-acetyl-L-cysteine (NAC) was applied to confirm its restoring effects on calcium metabolism and mineralization alteration in MC3T3-E1 cells. The finding of this study will contribute to explore the underlying mechanisms involved in the inhibitory potential of Aroclor 1254 on the differentiation process from pre-osteoblasts to mature osteoblasts.

## Materials and methods

### Cell culture and exposure

The mouse embryo osteoblast precursor MC3T3-E1 cells (Cell Bank of Academia Sinica, Shanghai, China), and α minimum essential medium (α-MEM) supplemented with fetal bovine serum (10%), penicillin (100 U/mL) and streptomycin (100 μg/mL) (Gibco, Grand Island, NY, USA) were purchased commercially. MC3T3-E1 cells were maintained and cultured in α-MEM at 37°C in a humidified atmosphere with 5% CO_2_. The standard of A1254 (AccuStandard, New Haven, CT, USA) was dissolved to 20 mmol/L in dimethyl sulfoxide (DMSO) (Sigma, St. Louis, MO, USA) and further diluted to 2.5, 5, 10 mmol/L with DMSO to prepare stocking solutions.

According to the internal exposure concentration of PCBs in human plasma (8) and the effective absorption rate (8.0-14.6%) of PCBs in cell model (29), the A1254 doses (2.5, 5, 10, and 20 μmol/L) were chosen in this study. The working solutions of A1254 were diluted using fresh cell culture medium with a final DMSO concentration of 0.1% (v/v). After treatment, the cell viability, ROS level, SOD activity, MDA level, calcium concentration, OCN content, calcium nodules and mRNA levels of related genes were measured. Moderate dose of A1254 (10 μmol/L) was reserved for the followed antioxidant experiments based on the results of cellular effects. MC3T3-E1 cells were co-incubated with both NAC (2 mmol/L, Sigma) and A1254 with a final DMSO concentration of 0.1% (v/v). The control group (Con) was treated with 0.1% DMSO/α-MEM (v/v) alone.All experiments were carried out at least three times with more than three parallel samples.

### Measurement of cell viability

Briefly, MC3T3-E1 cells were seeded in 96-well cell culture plates with a density of 3×10^3^ cells per well, and grown to exponential growth stage. Then, cells were incubated in 100 μL of fresh α-MEM medium with different concentrations of A1254 (0, 2.5, 5, 10, and 20 μmol/L) for 24h or 48h. After exposure, 10 μL of 3-(4,5-dimethyl-2-thiazolyl)-2,5-diphenyl tetrazolium bromide (MTT) in phosphate buffered solution (5 g/L) was added to culture medium and co-incubated at 37°C for another 4h. After that, the medium was discarded and 150 μL DMSO was added. The optical density values were measured and recorded using a plate reader (Multiscan Mk3, Thermofisher, MA, USA) at 490 nm.

### Oxidative stress detection

Production of intracellular ROS was assessed using a fluorescent probe 2’, 7’-dichlorofluorescein diacetates (DCFH-DA) (Beyotime, Wuhan, China). Briefly, cells were treated with A1254 (0, 2.5, 5, 10, and 20 μmol/L) alone or A1254 (10 μmol/L) + NAC (2 mmol/L) for 24h or 48h, with 0.1% DMSO (v/v) treatment as negative control. Then, MC3T3-E1 cells were incubated with 10 μmol/L of DCFH-DA for 30min. The fluorescence image was obtained using a microplate reader (Olympus, Tokyo, Japan) at an excitation 485 nm and emission 530 nm. The fluorescence intensity was quantitated using the Ipwin32 software (30).

SOD activity and MDA levels after A1254 exposure were determined using a SOD detection kit (S0101M, Beyotime, Shanghai, China) and MDA assay kit (S0131M, Beyotime, Shanghai, China), respectively. The SOD activity was calculated through colorimetric analysis of nitroblue tetrazoliumusing using a plate reader (Multiscan Mk3, Thermofisher, Massachusetts, USA) at 450 nm, and MDA levels were measured based on thiobarbituric acid chromogenic reaction using the Multiscan Mk3 plate reader at 535 nm.

### Measurement of calcium concentration

Fluorescent calcium indicator (Fluo 4-AM) (F14201, Thermofisher, Massachusetts, USA) was used to measure the intracellular calcium concentration. After treatment of A1254 (0, 2.5, 5, 10, and 20 μmol/L) alone or A1254 (10 μmol/L) + NAC (2 mmol/L) for 24h or 48h, MC3T3-E1 cells were incubated with 10 μmol/L of Fluo 4-AM for 30min. Fluorescence microscopy was conducted using a fluorescence microscope (Olympus, Tokyo, Japan) at 528 nm emission and 488 nm excitation. Data of fluorescence intensity was quantitated and analyzed using the Ipwin32 software (30).

### OCN content measurement

OCN enzyme linked immunosorbent assay (ELISA) Kit (YZ-E987967, Yanzun, Shanghai, China) was used to detect the OCN content in MC3T3-E1 cells according to the instructions. After treatment of A1254 (0, 2.5, 5, 10, and 20 μmol/L) alone or A1254 (10 μmol/L)+NAC (2 mmol/L) for 24h or 48h, the culture medium supernatant was collected. The absorbance at 450 nm was measured and recorded using the Multiscan Mk3 plate reader.

### Staining of calcium nodules

After treatment of A1254 (0, 2.5, 5, 10, and 20 μmol/L) alone or A1254 (10 μmol/L) + NAC (2 mmol/L) for 24h or 48h, MC3T3-E1 cells were incubated with 10 mmol/L of β-glycerophosphate and 10 nmol/L of dexamethasone (Sigma, Missouri, USA), and then further cultured for 14-28d until observation of visible calcium nodules formation. Then, MC3T3-E1 cells were fixed with alcohol (75%, v/v) and rinsed with phosphate buffered solution. Calcium nodules were stained with 40 mmol/L of Alizarin Red staining solution (Sigma, Missouri, USA), and the number of calcium nodules was counted.

### RT-qPCR assay

Transcription expression level of target genes related to osteoblastic differentiation was detected with RT-qPCR. Total RNA of MC3T3-E1 cells exposed to A1254 (0, 2.5, 5, 10, and 20 μmol/L) for 12h was extracted with TRIzol reagent (Invitrogen, California, USA). Then, reverse transcription of RNA samples was carried out using a qPCR RT Kit (18091050, Invitrogen, California, USA). Subsequently, RT-qPCR was performed with SYBR Green PCR Master Mix (Toyobo, Osaka, Japan) following the provided protocol. The amplification procedure was started at 95°C for 60 s, then followed by 50 cycles of denaturation at 95°C (15 s), annealing at 63°C (15 s), and extension at 72°C (45 s). RT-qPCR amplification reaction was conducted on a Bio-Rad iQ5 Real Time PCR (Bio-Rad, California, USA). The 2-ΔΔCt method was applied to calculate the fold changes of gene expression with Glyceraldehyde-3-phosphate dehydrogenase (*Gapdh*) as the calibrator. The primer sequences of *bsp*, *ocn*, *trpv6*, *alp*, and *Gapdh* were presented in **Table 1**.

**Table 1:** The primer sequence of target genes.

Genes	Orientation	Sequences (5’-3’)	Function of genes
Bone sialoprotein (*bsp*)	Forward Reverse	TCCATCGAAGAATCAAAGCAGAG CGAGAGTGTGGAAAGTGTGGAG	Anionic phosphoprotein involved in bone formation and mineralization.
Osteocalcin (*ocn*)	Forward Reverse	GGACCATCTTTCTGCTCACTCTG GTTCACTACCTTATTGCCCTCCTG	Marker of bone mineralization during remodeling process.
Transient receptor potential vanilloid 6 (*trpv6*)	Forward Reverse	CCCAAGCTTATTTTACTGAATTCT CGGGGTACCCTAGTAGGCCCAG	Epithelial ion channel mediating calcium influx and intracellular calcium content.
Alkaline phosphatase (*alp*)	Forward Reverse	GGACGGTGAACGGGAAAAT CTTCTCCACCGTGGGTCTCA	Marker of osteoblast differentiation involved in collagenous matrices *in vivo*.
Glyceraldehyde-3-phosphate dehydrogenase (*Gapdh*)	Forward Reverse	CCATGGAGAAGGCTGGGG CAAAAGTTGTCATGGATGACC	House-keeping glycolysis gene used as internal reference of gene expression

### Statistical analysis

Data were expressed as mean ± standard deviation of three independent experiments. Differences of results between groups were analyzed through one-way ANOVA and Turkey’s tests using a SPSS (13.0) software. A *p* value less than 0.05 was considered statistically significant.

## Results

### A1254 exposure induced cytotoxicity and oxidative stress in MC3T3-E1 cells

**Figure 1:**
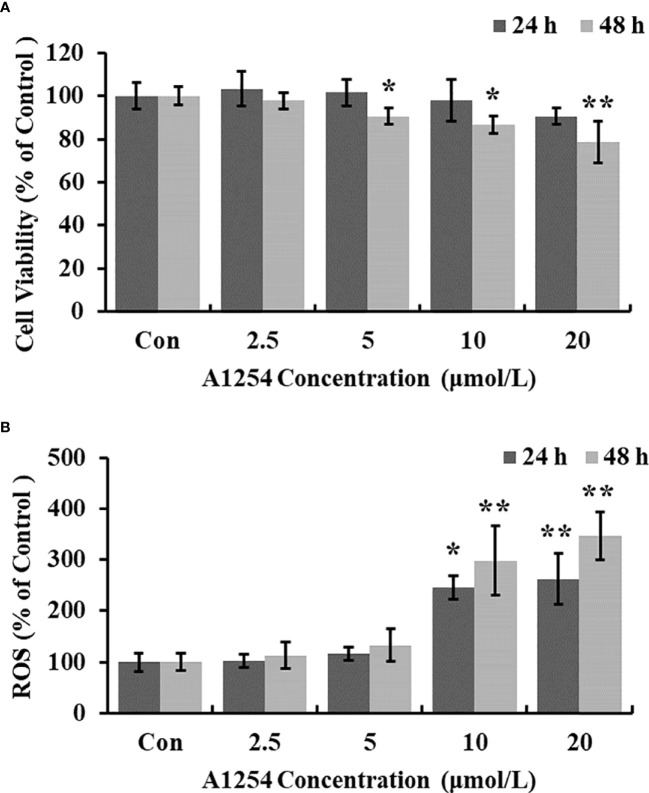
The cell viability and ROS level after A1254 exposure. MC3T3-E1 were incubated with various doses (0, 2.5, 5, 10, 20 μmol/L) of A1254 for 24h or 48h. **(A)** Cell survival was assayed by MTT assay. **(B)** ROS levels were determined with fluorescence microscopy using DCFH-DA probe. The fluorescent intensity was measured at 485 nm excitation and 530 nm emission, and analyzed using the Ipwin32 software. Each experiment was repeated three times with 8 replicates for each concentration group. *p<0.05, **p<0.01 compared to control.

**Figure 2:**
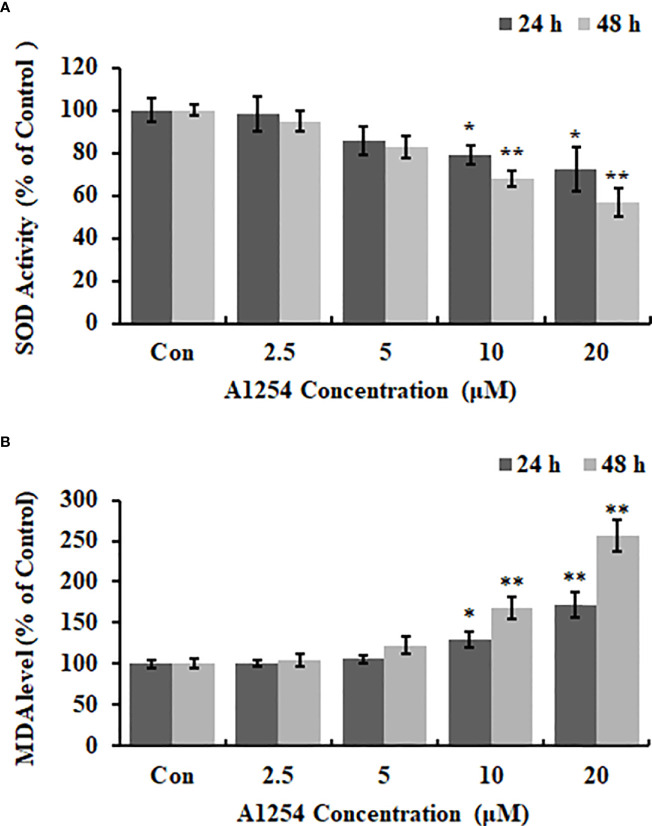
The SOD activity and MDA content after A1254 exposure. MC3T3-E1 were exposed to various doses of A1254 for 24 or 48h as described above. **(A)** SOD activity and **(B)** MDA content after A1254 exposure were detected using a SOD detection kit and MDA assay kit, respectively. Data derived from three independent experiments with 3 repeats for each group. *p<0.05, **p<0.01 compared to control.

Exposure of A1254 for 24h resulted in no obvious effects on cell viability of MC3T3-E1(*p*>0.05). After A1254 exposure for 48h, however, significant decrease of cell viability was observed (*p*<0.05, **Figure 1A**). After treatment of 10 and 20 μmol/L A1254 for 24h, the ROS level increased by 145% and 162%, respectively. And a 48-h exposure of A1254 (10 and 20 μmol/L) increased the ROS level by 198% and 247%, respectively, compared to the control group (*p*<0.05, **Figure 1B**). In addition, compared with control, the activity of SOD significantly reduced and the MDA level significantly increased with time- and concentration-dependent manner, after 10 and 20 μmol/L of A1254 treatment for 24 and 48h (*p*<0.05, **Figure 2**). In general, exposure of A1254 induced significant oxidative stress in MC3T3-E1 cells, which might be associated with consequent reduction of cell viability.

### A1254 reduced the intracellular calcium and mineralization level

Treatment of 10 and 20 μmol/L of A1254 for 24 and 48h significantly reduced the intracellular calcium levels (*p*<0.05, **Figuress 3A, B**). Furthermore, the OCN content was significantly lower in the A1254 exposure groups (10 and 20 μmol/L, 24 and 48h) than that of the control group after time-points analysis; and the nodule formation rate was significantly decreased (*p*<0.05, **Figures 4A, B**).

**Figure 3:**
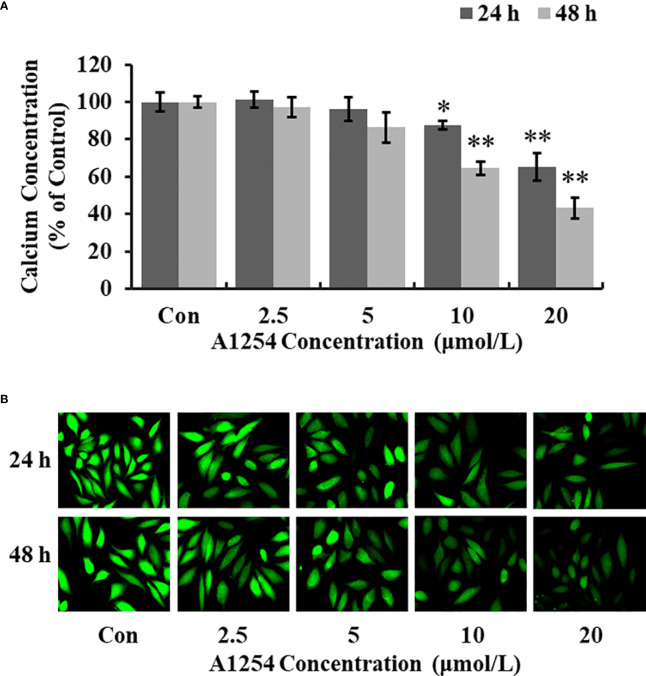
The calcium concentration in MC3T3-E1 cells affected by A1254. MC3T3-E1 were exposed to various doses of A1254 for 24 and 48h. The calcium concentration was assessed with fluorescence microscopy using a Fluo 4-AM probe. Fluorescent intensity was measured and recorded at 528 nm emission and 488 nm excitation; and data were analyzed using the Ipwin32 software. **(A)** Relative intracellular calcium levels compared to control. **(B)** Representative fluorescence images. Each experiment was repeated three times. *p<0.05, **p<0.01 compared to the control.

**Figure 4:**
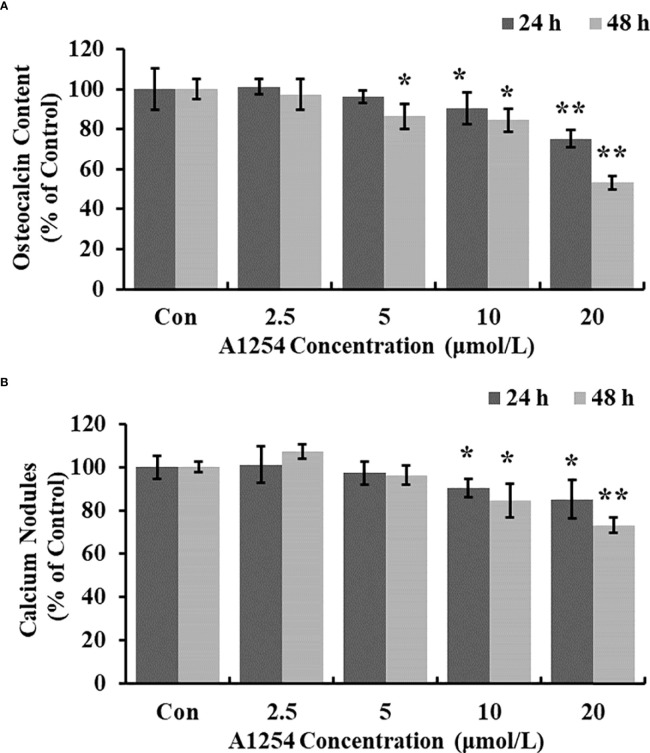
The osteocalcin content and calcium nodules formation influenced by A1254. After exposure to various doses of A1254 for 24 and 48h, **(A)** The osteocalcin content in MC3T3-E1 cells was assessed with ELISA kit. **(B)** The calcium nodules formation was detected using Alizarin Red staining. The number of calcium nodules was counted, and nodules formation rate was calculated compared with the control group. *p<0.05, **p<0.01 compared to the control.

### A1254 reduced expression of marker genes of osteoblasts differentiation and bone formation

**Figure 5:**
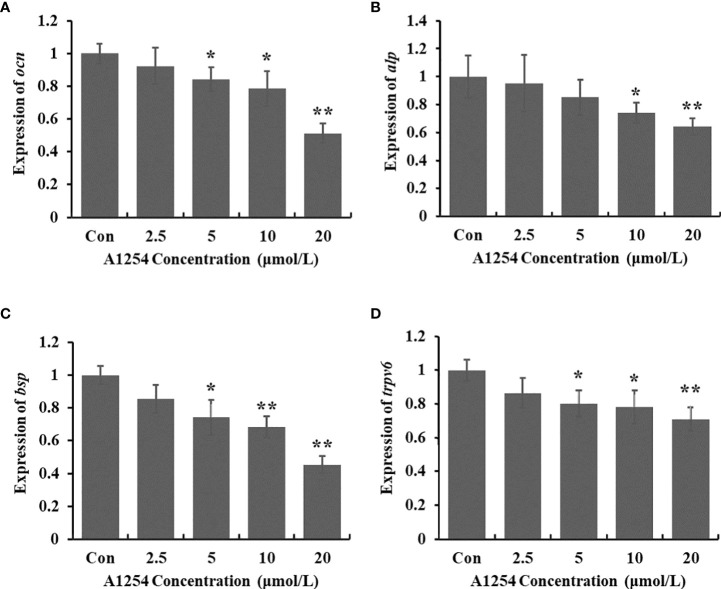
The expression of bone turnover markers impaired by A1254 exposure. MC3T3-E1 cells were incubated with various doses of A1254 for 12h, and expressions of **(A)** *ocn*, **(B)** *alp*, **(C)** *bsp* and **(D)** *trpv6* were analyzed with RT-qPCR. Fold changes of gene expression was calculated using the 2-ΔΔCt method, with *Gapdh* as an internal reference. *p<0.05, **p<0.01 compared to the control.

To clarify the inhibitory effects of A1254 on osteoblast differentiation, the transcriptional expression levels of target genes related to bone turnover such as *bsp*, *ocn*, *trpv6*, and *alp* were analyzed with RT-qPCR. A1254 exposure (5, 10 and 20 μmol/L) significantly reduced the *ocn* expression with a 15.9%, 21.5%, and 48.8% decrease, respectively (*p*<0.05, **Figure 5A**). After 10 and 20 μmol/L of A1254 exposure for 12h, the expression of *alp* reduced by 25.8% and 35.8%, respectively (*p*<0.05, **Figure 5B**). In addition, the expression of *bsp* was obviously reduced after A1254 exposure with 14.6-54.8% reduction (*p*<0.05, **Figure 5C**). The expression of *trpv6* was also significantly decreased after A1254 exposure (10 and 20 μmol/L) with a 13.5% to 29.0% reduction (*p*<0.05, **Figure 5D**). Compared to the control group, the decreased expression of *bsp*, *ocn*, *trpv6*, and *alp* genes indicated that osteoblast differentiation may be impaired by A1254 exposure through reduction of bone mass.

### NAC partially restored A1254-induced decrease on osteoblast differentiation

**Figure 6:**
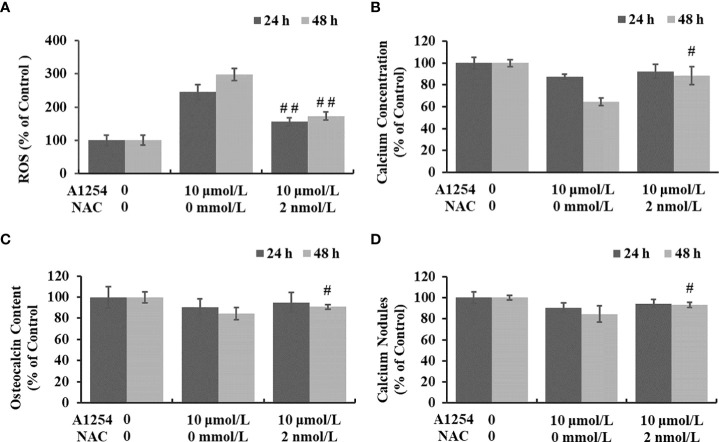
NAC partially relieved the osteotoxicity induced by A1254 exposure. MC3T3-E1 cells were treated with A1254 (10 μmol/L) alone or NAC (2 mmol/L) +A1254 (10 μmol/L) for 24 and 48h. ROS production, calcium level, osteocalcin content, and calcium nodules formation rate were measured. Data between NAC group and non-NAC group were compared with one-way ANOVA. **(A)** Relative ROS level after exposure. **(B)** The calcium level measured with Fluo 4-AM probe. **(C)** The osteocalcin content detected with ELISA kit. **(D)** The calcium nodules formation rate compared to control detected using Alizarin Red staining. #p<0.05, ##p<0.01 compared to non-NAC group.

NAC tests were performed to confirm the role of oxidative stress on osteotoxicity induced by A1254 exposure, and the intracellular calcium level, OCN content and nodules formation were assayed and compared between NAC groups and non-NAC groups. Compared with A1254 (10 μmol/L) treated group (non-NAC group), the ROS level of NAC group reduced 36.4% and 42.0% after 24 and 48h exposure, respectively (*p*<0.05, **Figure 6A**). As shown in **Figures 6B-D**, after co-incubation of NAC (2 mmol/L)+A1254 (10 μmol/L) for 48h, the calcium level, OCN content and nodules formation rate increased 36.9%, 7.6%, and 10.4%, respectively, compared to the non-NAC group (*p*<0.05). These findings demonstrated that oxidative stress did play a role in reduction of osteogenesis and osteoblast differentiation induced by A1254 exposure in MC3T3-E1 cells.

## Discussion

Previous research has indicated that PCB exposure suppressed the differentiation of osteoblasts (31). Recently, several studies were conducted to explore the molecular mechanisms involved in PCB-induced bone toxicity. A prenatal exposure of PCB 180 to pregnant rats resulted in alteration of liver retinoid level, increased 7-pentoxyresorufin O-dealkylase activity, and induction of cytochrome P450 enzymes in offspring, indicating that the constitutive androstane receptor (CAR) or pregnane-X receptor (PXR) pathways may participate in impaired bone maturation processes (32). Herlin etal. (33) found that impact of 2,3,7,8-tetrachlorodibenzo-p-dioxin on bone quality of mice was closely associated with increased cytosolic retinoic acid through repressed expression of cytochrome P450 enzyme 26a1 and cellular retinoic acid-binding protein type 2 in the osteoblasts, and induction of aryl-hydrocarbon receptor (AHR) repressor, cytochrome P450 enzymes 1b1 and 1a1 expressions. It is well established that some PCB-congeners bind to and alter the AHR, CAR and/or PXR pathways in the cell, leading to induction of oxidative stress and various adverse effects (34).

In the present study, significant inhibition of cell viability was found in MC3T3-E1 cells exposed to A1254 exposure for 48h. The previous results of An etal. (30) also showed that A1254 reduced the cell viability and induced cell apoptosis of osteoblasts, which was consistent with our results (**Figure 1A**). PCB has a high-affinity binding site for aryl hydrocarbon receptors, which can mediate ROS production and antioxidant scavenge, disturbing cellular redox balance (35, 36). Alterations of the redox status are closely related to A1254-induced bone remodeling disorders and pathogenesis of bone loss (37, 38). Reduction of excess oxidative stress by melatonin could effectively improve periprosthetic bone mass and initial implant stability during bone remodeling process in ovariectomized rats (39). In this study, results of intracellular ROS production displayed that A1254 significantly promoted ROS accumulation in a dose-dependent manner in MC3T3-E1 cells.

SOD is known as a key antioxidant involved in the cellular defense system against excessive free radicals (40). As a product of lipid peroxidation, excessive MDA production leads to dysfunction of biological macromolecules such as proteins and nucleic acids, which can finally induce cytotoxic effects (41). Excessive oxidative stress was proven to play a vital role in impaired osteoblasts differentiation and bone loss, which can be relieved by antioxidants through their direct or indirect counteracting effects with oxidants (38). In this study, induction of ROS production, excessive MDA generation and reduction of SOD activity after A1254 exposure indicated that oxidative stress was involved in the osteotoxicity induced by A1254.

It has been documented that the intracellular calcium could affect the crosstalk between osteoclasts differentiation and osteoblasts formation (X. 42–44). Williams A. E. etal. (45) showed that PCB126 reduced serum calcium in rats, resulting in shorter tibia length, and smaller cortical/medullary area. PCB118 activated osteoclasts and induced the bone resorption process in goldfish (46). In this study, we also found that the level of intracellular calcium was significantly reduced in MC3T3-E1 cells exposed to A1254 (**Figures 3A, B**), which may consequently influence the osteogenesis process and bone mass loss. The OCN content detected in this study was markedly lowered in the A1254 exposure groups (**Figure 4A**). The repressing effect of A1254 on OCN consequently decelerated the formation of mineralization nodules in MC3T3-E1 cells (**Figure 4B**), which is consistent with previous research of Herlin etal. (31).

Furthermore, process of osteoblast differentiation and formation was regulated through expression of some related genes such as *ocn*, *alp*, *bsp*, and *trpv6.* Reduced expression level of *ocn* gene was consistent with the significant reduction of OCN content in this study. Expressions of *alp* and *bsp* were significantly decreased after A1254 exposure. Previous studies also found that 2,3,7,8-tetrachlorodibenzo-p-dioxin induced cytotoxicity in MC3T3-E1 cells and reduced the expressions of *alp* and *bsp* (47, 48). Reduction on expression of *ocn*, alp, and *bsp* suggested that the inhibitory effects of A1254 on osteoblasts differentiation and bone formation might result from deregulation of related target genes after A1254 exposure. It has been reported that A1254 reduced the cellular calcium level by down-regulation of TRPV6 in osteoblasts (30). The reduction of *trpv6* expression after exposure indicated the impact of A1254 on calcium homeostasis, osteoblast differentiation and bone formation (**Figure 5D**).

Generally, NAC acts as a ROS scavenger to protect organisms against peroxidative stress damage and possibly modulates the osteoblastic differentiation (49). Our results showed that NAC decreased the induction of oxidative stress by A1254 and significantly reduced ROS accumulation (**Figure 6A**). Moreover, the calcium concentration and bone mineralization were also partially restored (**Figures 6B-D**). These results suggested that oxidative stress induction was involved in osteotoxicity induced by A1254. Further in-depth studies on the signal pathways related to oxidative stress are needed to be conducted to clarify the detailed mechanism of PCB-induced osteotoxicity.

## Conclusions

**Figure 7:**
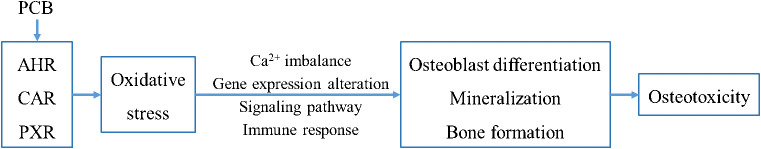
The schematic diagram of A1254-induced osteotoxicity.

As shown in the schematic diagram (**Figure 7**), A1254 dose-dependently reduced the cell viability in MC3T3-E1, and induce oxidative stress through increase of intracellular ROS level and MDA content, and reduction of SOD activity. The reduction of intracellular calcium level, OCN content and nodules formation rate, as well as decreased expression of relative markers for bone turnover (*ocn*, *alp*, *bsp*, and *trpv6*) suggested the impact of A1254 on calcium homeostasis and osteoblast differentiation. Moreover, partial restoration on redox status, calcium metabolism, and mineralization inhibition by antioxidant NAC indicated that oxidative stress plays a crucial role in A1254-induced ototoxicity.

## Data availability statement

The original contributions presented in the study are included in the article/Supplementary Material. Further inquiries can be directed to the corresponding authors.

## Author contributions

SX and YC designed research. YWC, CC, ML, LL performed research. ZY, SW and LF performed data analysis. YC, LF and SX wrote the manuscript. All others read, edited, and approved the manuscript. All authors contributed to the article and approved the submitted version.

## Conflict of interest

The authors declare that the research was conducted in the absence of any commercial or financial relationships that could be construed as a potential conflict of interest.

## Publisher’s note

All claims expressed in this article are solely those of the authors and do not necessarily represent those of their affiliated organizations, or those of the publisher, the editors and the reviewers. Any product that may be evaluated in this article, or claim that may be made by its manufacturer, is not guaranteed or endorsed by the publisher.
